# Exploring Relationships between Host Genome and Microbiome: New Insights from Genome-Wide Association Studies

**DOI:** 10.3389/fmicb.2016.01611

**Published:** 2016-10-12

**Authors:** Muslihudeen A. Abdul-Aziz, Alan Cooper, Laura S. Weyrich

**Affiliations:** Australian Centre for Ancient DNA, School of Biological Sciences and The Environment Institute, The University of Adelaide, AdelaideSA, Australia

**Keywords:** microbiome, GWAS, ancient DNA, microbiome GWAS, model organisms, microbiota, symbiosis

## Abstract

As our understanding of the human microbiome expands, impacts on health and disease continue to be revealed. Alterations in the microbiome can result in dysbiosis, which has now been linked to subsequent autoimmune and metabolic diseases, highlighting the need to identify factors that shape the microbiome. Research has identified that the composition and functions of the human microbiome can be influenced by diet, age, sex, and environment. More recently, studies have explored how human genetic variation may also influence the microbiome. Here, we review several recent analytical advances in this new research area, including those that use genome-wide association studies to examine host genome–microbiome interactions, while controlling for the influence of other factors. We find that current research is limited by small sample sizes, lack of cohort replication, and insufficient confirmatory mechanistic studies. In addition, we discuss the importance of understanding long-term interactions between the host genome and microbiome, as well as the potential impacts of disrupting this relationship, and explore new research avenues that may provide information about the co-evolutionary history of humans and their microorganisms.

## Introduction

Our view of the microbial community within us has shifted drastically in the last few decades from simplistic commensalism to complex mutualisms. As a comprehensive understanding of this microbial community expands, we are beginning to unravel the impact of microbes on human health. This diverse microbial community is defined as the ‘microbiota,’ and the collective genomes of all microbial species and their environment is termed the ‘microbiome’ (Marchesi and Ravel, 2015). Microbiota play key roles in human health throughout life (Gregory, 2011; Yatsunenko et al., 2012; Rodríguez et al., 2015; Zapata and Quagliarello, 2015), and dysbiosis, defined as an imbalance of the composition of the microbial components of the microbiota (Karlsson et al., 2013; Parekh et al., 2015) has now been linked with various metabolic diseases such as obesity, type 2 diabetes and inflammatory bowel disease (IBD).

Our improved ability to examine the microbiome is a result of a revolution in DNA sequencing technologies and techniques, and the reapplication of pre-existing concepts from ecology. Exponential advances in DNA sequencing technology, from low-throughput Sanger sequencing to high-throughput next generation sequencing, have allowed us to obtain huge amounts of sequence data at a fraction of the cost (Cho and Blaser, 2012; van Dijk et al., 2014). Furthermore, the availability of cheap computational power, coupled with free and readily available open source software tools, has resulted in larger capacities for in-depth analysis of sequenced data to characterize microbial communities. In addition, the applications of concepts from ecology, such as diversity indices [Alpha diversity and Beta diversity (Whittaker, 1972)], have enabled us to better categorize and understand the composition and diversity of the microbial world within us (Blaser, 2014).

Two separate methods are commonly applied to examine human microbiota: metabarcoding or shotgun sequencing. Metabarcoding typically uses the 16S ribosomal RNA (rRNA) encoding gene to characterize the species structure of bacterial communities in various environments, and has been the gold standard in microbiome research due to the ubiquity of the 16S rRNA gene amongst prokaryotes and the availability of large reference databases (Woese et al., 1990). However, shotgun metagenomic sequencing, or sequencing DNA fragments at random from the sample, is increasing in popularity, as it can be used for both species profiling and functional analysis. Both approaches have limitations. For example, 16S rRNA sequencing can be biased due to uneven amplification of bacterial 16S rRNA genes or primer biases. While challenges with identifying low abundance community members with shotgun metagenomic sequencing and the cost of large-scale multiplexing at sufficiently high depths make this method prohibitive for many labs, shotgun metagenomic sequencing has the potential to provide information about the microbial community and the host simultaneously (Poretsky et al., 2014).

From an evolutionary perspective, microbiome studies have revealed compositional differences in microbiota between great apes, archaic hominins, and modern humans, with a marked reduction in microbial diversity observed within modern humans. This loss of bacterial diversity in modern humans is postulated to be a result of lifestyle changes (Cho and Blaser, 2012). Dietary changes brought about by agriculture altered the human microbiota considerably over 7,500 years ago, and changed again with the recent movement toward animal-based and fat-rich western diets (Adler et al., 2013). This reduction in diversity is thought to be partially responsible for the dysbiosis observed in modern human microbiomes that is now associated with various metabolic and autoimmune diseases (Blaser and Falkow, 2009; Adler et al., 2013; Moeller et al., 2014; Logan et al., 2016). Furthermore, changes in the human microbiome through time and the linked impacts on human health highlight the importance of studying the co-evolution of the human microbiome and how those evolutionary changes in microbial composition may have interacted with our genomes (Linderholm, 2015).

In parallel with these recent discoveries in microbiome research, the last two decades of genomic research have expanded our understanding of how human genomic differences result in phenotypic changes that impact human health and well-being. The advent of large genome wide association studies (GWAS) on a population level have allowed us to better understand the relationships between common genetic variants and diseases, such as Alzheimer’s, type 2 diabetes and obesity (Imamura and Maeda, 2011; Fall and Ingelsson, 2014; Rao et al., 2014). However, these two research fields, microbiome research and human population genetics, have only recently begun to explore the human genome and microbiome simultaneously, to examine how their interaction influences our health and disease. Our understanding of their interactive roles in our health and disease remains in its infancy (Gilbert et al., 2016; Wang and Jia, 2016). This review highlights in chronological order advances linking host genetic variation with microbiome composition and more recent research using GWAS.

## Microbiomes

The Human Microbiome Project (HMP) determined that different body sites are distinct niches for various bacteria, resulting in differences in microbiome composition throughout the body (The Human Microbiome Project, 2012). The human gut, due to its large surface area and role in nutrition and energy homeostasis, is home to a plethora of microbes. Its microbiome has been the most studied due to its easy access for sampling and critical importance for health. The gut microbiome has been shown to play essential roles in the metabolism of complex polysaccharides. These complex polysaccharides are used to synthesize short-chain fatty acids such as butyrate, acetate, and propionate that are used as signaling molecules in the communication and development of host innate immune system (Jacobs and Braun, 2014). This system can breakdown due to changes in microbiome composition or pathogenic microorganism colonization that hijacks and alters these pathways, contributing to the etiology of metabolic and infectious diseases, such as type 2 diabetes, IBD, obesity (Carding et al., 2015), and *Clostridium difficile* infection (CDI; Bien et al., 2013). In addition to the influence of the gut microbiome on the immune system, short-chain fatty acids produced by the gut microbiota have also been shown to impact the brain and nervous system (Rhee et al., 2009; Thomas et al., 2012).

Microbiomes at different body sites interact and can influence one another. This interaction between microbiomes at different sites in the body, such as the oral and gut microbiomes, was indicated in a study by Ding and Schloss (2014) where specific microbes abundant in the gut were more likely to be present in the oral cavity (saliva and supragingival plaque). This predictive relationship between the oral and gut microbiota is not surprising, as the oral cavity is the gateway to the gut for various microbes (Dewhirst et al., 2010). The advent of modern dentistry has exposed the presence of pathogenic bacteria in the oral cavity. However, our understanding of the role played by non-pathogenic commensal bacteria is relatively recent. Recent research has revealed over 600 bacterial species or phylotypes present at different sites in the oral cavity (Dewhirst et al., 2010), including many commensal species that help in host defense by colonizing prime locations and creating an inhospitable environment for secondary colonization of pathogenic bacteria. Disruption of the composition of this healthy oral microbiome has been observed to result in inflammation and diseases such as periodontitis and the proliferation of bacterial species, such as *Streptococcus mutans*, which play crucial roles in the etiology of dental caries (cavities) (Johansson et al., 2015; Kumar and Mason, 2015). This is exemplified by recent research showing that the oral microbiomes of patients with dental caries are distinct in composition and abundance of *Streptococcus* compared to that of healthy individuals (Johansson et al., 2015).

In order to study host genome and microbiome interactions, it is crucial to examine other factors that influence microbiome composition. Recent advances in microbiome research has shown that microbial composition can be heavily influenced by the environment through diet and lifestyle (David et al., 2014). Western populations have homogeneous diets and lifestyles resulting in lower microbial diversity. Microbiomes from non-western and indigenous populations are currently being explored as a means to examine how unique lifestyles impact the human microbiome. Studies examining microbiota in African hunter-gatherers such as the Hadza (Schnorr et al., 2014) and South American indigenous peoples (Yatsunenko et al., 2012) have revealed a broader picture of the human gut microbiome – one that is more diverse and complex. Sex has also been shown to influence microbial composition with a number of recent studies correlating specific microbial composition to specific sexes (Markle et al., 2013; Ding and Schloss, 2014; Blekhman et al., 2015). In many studies, such as those on the microbiomes of the Hadza and Hutterites (a North American isolated community with shared diet and non-standard cultural practices), differences in microbial compositions between the sexes are related to the dissimilarities in societal roles played by men and women (Schnorr et al., 2014; Davenport et al., 2014, 2015). Age is another factor that influences microbial composition. In infants, the gut microbiome is highly unstable and can resemble that of the mother, but shifts toward a more robust adult microbiome within 2–3 years of life (Yatsunenko et al., 2012). This shift may be due to changing diets due to the increase in age. In a murine model system, Langille et al. (2014) demonstrated differences in microbial composition between old and young mice, and identified specific bacterial genera, such as *Alistipes*, that were more prevalent in older mice. Furthermore, two recent publications using large datasets (*N* > 1000) confirmed these factors, as well as 69 others that fall in categories, such as medication, blood parameters, health status, anthropometric features, and lifestyle that correlated with variation in microbiome composition (Falony et al., 2016; Zhernakova et al., 2016). These differences in age, sex, populations, culture, and environment will need to be addressed in studies exploring human genome–microbiome interactions.

## Exploring Host Genome–Microbiome Interactions

### Animal Models

Animal models provide an avenue to probe these interactions at a depth that is not possible using human-based studies. Studies using zebrafish (Kanther and Rawls, 2010; Milligan-Myhre et al., 2011), *Drosophila* (Kuraishi et al., 2013), and mice have been crucial for laying the foundations of microbiome research (Kostic et al., 2013). Many early animal-based microbiome studies focused on the symbiosis between microbial communities and their hosts. Sharon et al. (2010) illustrated the role that commensal microbiota play in the mating preference of *Drosophila melanogaster*. In zebrafish, the influence of the host genome on the microbiome was confirmed by an experiment involving transplantation of microbiota between germ-free zebrafish and mice. The transplanted microbiota adapted to resemble the host’s normal microbiota, demonstrating that the host genome selects for specific microbes to suit certain niches within an organism (Rawls et al., 2006).

### Murine Models

Microbiome research in mice has been ground-breaking, as germ-free mice have played crucial roles in many baseline experiments. Murine models have shown that gut microbiota are influenced by the genetic background of the mice (Esworthy et al., 2010), and that gut microbial composition should be viewed as a complex polygenic trait (Benson et al., 2010). Mice have a large number of orthologous genes and similarities in microbiome composition to humans, and quantitative trait loci (QTL) analyses in mice have revealed specific gene regions that modulate microbiome composition (Srinivas et al., 2013). Benson et al. (2010) reported 18 QTLs that were associated with specific bacterial taxa. Furthermore, McKnite et al. (2012) used genetic mapping to link gut microbiota of laboratory crossed mice to immune genes that modulate microbiome composition.

### Human-Based Studies

Murine model studies have consistently shown that host genotypes are important in regulating microbiota composition (Campbell et al., 2012; Hildebrand et al., 2013; Bongers et al., 2014). While murine models demonstrate the importance of genotypes in regulating the composition of microbiota, they cannot be viewed as replacements for human-based studies due to the inherent complexity involved in extrapolating murine results to humans (Seok et al., 2013). Turnbaugh et al. (2009) and Yatsunenko et al. (2012) used dizygotic and monozygotic twins to examine the heritability of the gut microbiome while controlling for environmental factors. Although both studies concluded that there were no statistically significant differences between monozygotic and dizygotic twins, they were both underpowered due to their small sample sizes and lack of statistical rigor. Consequently, a follow up studies using larger sample sizes of the same twin datasets by Goodrich et al. (2014) and more recently by Goodrich et al. (2016a) revealed the measurable influence of host genetic variation on microbial composition, showing *Christensenellaceae* to be the most heritable taxon, while *Bacteroidetes* was more influenced by the environment. Goodrich et al. (2016a) also showed that highly heritable taxa correlated with higher levels of temporal stability, highlighting the importance of these taxa to the host.

A candidate gene approach has also been explored (Jacobs and Braun, 2014). Research by Folseraas et al. (2012) and Knights et al. (2014) examined the role of gut microbiome composition and host genetic loci associated with IBD and related diseases in humans. Folseraas et al. (2012) showed that the gene FUT2 is associated with a signficant increase in the abundance of Firmicutes and significant decrease of Proteobacteria, while Knights et al. (2014) correlated the NOD2 gene with Enterobactericea, pointing toward the impact of these two genotypes and their associated bacterial taxa as risk factors for IBD and IBD related diseases.

While these studies provided valuable insight into how the microbiome can be inherited like other phenotypic traits, their study design using heritability is limited, as it cannot identify statistically significant genetic loci on a genome-wide level that interact with gut microbiota composition.

### The Advent of Microbiome – GWAS Research

Recently, researchers have begun to apply techniques from GWAS to microbiome research. GWAS seek to identify relevant common genetic variants associated with various disease phenotypes, by simultaneously screening thousands of the most common variants in the human genome and these phenotypes (Bush and Moore, 2012). In humans, GWAS have successfully linked genetic variation to various disease phenotypes, including type 2 diabetes, obesity, and heart disease (Visscher et al., 2012). However, very few studies have used GWAS to explore the interaction between human genetics and microbiome composition.

Three recent studies have broke new ground by using GWAS to study the influence of genetic variation on microbiome composition. Blekhman et al. (2015) reported the first microbiome GWAS study based on genomic and microbiome data mined from the HMP. Microbiome data from 15 sites on the body were correlated with human genomic data inferred from “contaminating” human DNA of the body sites to expose the role that host genetic variation plays in microbiome composition across various sites in the body. These inferences on the host were made possible due to the large amounts of host DNA found, which were variable at different body sites. A subsequent publication by Davenport et al. (2015) analyzed the gut microbiome and host genetic data from Hutterites, a North American isolated community with shared diet and identical cultural practices, to control for environmental confounders. The third and most recent study by Goodrich et al. (2016a) revisited the inheritance of the gut microbiota in twins and examined the link between host genetics and gut microbiome composition in 1,248 individuals, including mono- and di-zygotic twin pairs. All three studies (Blekhman et al., 2015; Davenport et al., 2015; Goodrich et al., 2016a) revealed that specific bacterial taxa, such as *Bifidobacterium*, are inheritable and correlate with specific host genotypes. Goodrich et al. (2016a) linked *Bifidobacteria* which metabolizes lactose in the gut with the LCT gene locus which encodes for the enzyme lactase that hydrolyses lactose. They revealed that lactase ‘non-persisters’ harbored lower levels of *Bifidobacterium* relative to lactase ‘persisters,’ possibly due to the higher availability of lactose in the gut of lactase non-persisters. Blekhman et al. (2015) and Davenport et al. (2015) also found that immunity-related genes, such as interleukin-2 (IL2) influenced the modulation of microbiome composition via pathways that may then result in complex diseases, although the exact mechanism remain unknown. Goodrich et al. (2016a) also updated and summarized an earlier list by Spor et al. (2011) of number of other host genetic loci that are suspected to be associated with the microbiome that was primarily based on mouse QTL research.

Furthermore, these studies used gene expression data to illustrate that genetic loci linked to microbiome composition have a functional role in metabolic diseases, such as obesity (Blekhman et al., 2015; Davenport et al., 2015).

### Microbiome – GWAS Methodology

Thus far, many of the most recent microbiome GWAS studies on human hosts used either Alpha diversity indices (α-diversity; within population diversity) or Beta diversity indices (β-diversity; between population diversity) of microbial composition as phenotypes, and correlated the diversity with common genetic variants (SNPs). **Table 1** details the methods used in microbiome GWAS in human hosts published to date, based on our knowledge. Blekhman et al. (2015) utilized a traditional GWAS statistical software package, PLINK (Purcell et al., 2007), and completed regression analysis with an additive linear mixed model, followed by multiple test corrections to identify SNPs associated with microbiome composition at genome-wide significances. However, Davenport et al. (2015) and Goodrich et al. (2016a) used a genome-wide efficient mixed model implemented in the GEMMA software tool in their studies (Zhou and Stephens, 2012). These studies demonstrated that β-diversity is a more appropriate metric than α-diversity for microbiome GWAS, as it represents the microbial community more comprehensively and reduces the temporal variability observed with α-diversity resulting in an increase in statistical power (Hua et al., 2015). Microbiome GWAS (Hua et al., 2015) is a recently developed statistical software package that uses β-diversity indices calculated using both unweighted or weighted UniFrac and genome-wide SNPs, and allows for controlling confounders as covariates in a computationally efficient framework. Microbiome GWAS has been tested using both simulations and on a lung cancer dataset to identify microbiota associated with lung cancer risk in Europeans (Hua et al., 2015). More recently, Microbiome GWAS was applied to the expanded UK twins dataset (*n* = 1,248) (Goodrich et al., 2016a). This statistical package has the capability to correct for skewness and kurtosis in the score statistics that are a result of small sample sizes. Another recently published software package is MiRKAT (Zhao et al., 2015), which is a kernel regression based test to find associations between genome-wide SNPs and β-diversity computed using a generalized UniFrac (Chen et al., 2012). MiRKAT is limited by its computationally inefficient framework, and requires long run time and a large amount of processing power (Hua et al., 2015).

**Table 1:** Methods used in human microbiome Genome wide association studies (GWAS).

Method	Software tool	Sample size	Microbiome (sampling site)	Number of SNP/Genes correlated	Reference	Notes
Additive linear mixed model	PLINK (v1.07)	93	Microbiome composition at various body sites (10)	83^∗^	Blekhman et al., 2015	^∗^Pathway based analysis was used in lieu of genes.
	Purcell et al., 2007;					
	Chang et al., 2015					
Genome-wide efficient mixed-model association (GEMMA)	GEMMA (v0.94)	127	Gut microbiome	187^∗^	Davenport et al., 2015	^∗^SNP
	Zhou and Stephens, 2012					
Microbiome GWAS	Microbiome GWAS	1,248	Gut microbiome	142^∗^	Goodrich et al., 2016a	^∗^Genes
	Hua et al., 2015					^∗^GEMMA (v0.94) was also used.


These microbiome – GWAS wide association studies form the initial attempts to elucidate genome–microbiome interaction; however, a number of limitations currently exist. GWAS typically require large sample sizes in order to obtain statistically significant results and account for small effect sizes that can be attributed to variants correlated with phenotypes (Hayes, 2013). The studies of Blekhman et al. (2015) and Davenport et al. (2015) both had small sample sizes (∼100) that likely resulted in underpowered statistical analyses, especially following the multiple test corrections that were required for the results to be statistically significant. Blekhman et al.(2015) combined various individual SNPs into groups based on their association with similar biological pathways to increase statistical power, while Davenport et al. (2015) performed multiple test corrections on SNPs within each genome-wide association study to reduce the number of SNPs undergoing multiple test corrections. Nevertheless, both of these approaches do not fit the required statistical rigor observed in traditional GWAS studies (Barsh et al., 2012). Another challenge in microbiome – GWAS studies is the need for replicate cohorts. While Blekhman et al. (2015) used the Twins UK dataset (Moayyeri et al., 2012), Davenport et al. (2015) lacked a replication cohort to confirm their results. Improving statistical power by accounting for excess zeros in OTU counts of metagenomic samples (Xu et al., 2015), as well as larger host sample sizes and replication cohorts, are crucial if we are to obtain a more reliable understanding of genome–microbiome interactions. Furthermore, while most studies of this type identify genetic loci that appear to interact with the genome, all are yet to be confirmed by mechanistic or functional studies.

## Evolutionary Perspective on Genome–Microbiome Interactions

Due to changes in the human microbiome and genome over long periods of time, an evolutionary perspective on the microbiome is important in interpreting these effects on human health and disease. Several groups have examined modern populations to infer the influences of different evolutionary histories on the human microbiome. Moeller et al. (2014) showed how the human microbiome evolved by sequencing the gut microbiome of chimpanzees, bonobos, gorillas, and modern humans. More recently, they reported the presence of shared microbial composition across all four host species, suggesting the existence of an ancestral hominid microbiome (Moeller et al., 2016). In addition, Moeller et al. (2014) showed that the microbiome of our hominid ancestors was more diverse and more stable compared to that of modern humans. Disruptive dietary and environmental changes, such as the transition to agriculture, may have likely contributed to the loss of such diversity (Adler et al., 2011). Moeller et al. (2014) observed that bacterial taxa, such as *Bacteroidetes*, are associated with animal fat, rich diets, and high protein intake, and increase in abundance in modern humans. In contrast, taxa responsible for the degradation of complex polysaccharides, such as *Methanobrevibacter*, underwent decreases in abundance. The authors also compared their data to people in remote Venezuela, rural Malawi, and industrial United States, demonstrating a gradient of diminished microbiotic diversity driven by environment and diet (Moeller et al., 2014, 2016).

Similar findings have also been observed in other studies, Schnorr et al. (2014) and Schnorr (2015) explored the gut microbiome of the Hadza people, an Indigenous group of Tanzanian Hunter-gatherers. Researchers observed high bacterial diversity in this group compared to Western populations, as well as higher abundances of plant polysaccharide metabolizing taxa, such as *Prevotella* and *Treponema*. These taxa likely provide the Hadza with short chain fatty acids, such as butyrate and propionic acid, which positively influence immune and nervous systems by regulating inflammation (Rhee et al., 2009; Thomas et al., 2012). Other researchers hypothesized that the diversity and composition of the Hadza’s microbiome contribute to their low rates of nutritional and metabolic diseases (Blurton Jones et al., 1992). Similarly, De Filippo et al. (2010) and Yatsunenko et al. (2012) have shown the presence of a decreasing gradient of microbial diversity and abundance from contemporary Hunter-gatherers, rural farming communities to western populations. These studies suggest that our changing diets and lifestyles are responsible for our “missing microbes,” and the resulting metabolic and autoimmune diseases (Blaser and Falkow, 2009).

### Ancient DNA

Expanding the focus of microbiome studies to non-western and indigenous populations around the world has helped us to understand the diversity of the human microbiome. However, these studies do not provide us the information necessary to understand evolutionary changes through time. The expansion of microbiome research from contemporary to ancient human populations has provided us with evolutionary snapshots of change through time, allowing for the examination of the human microbiome before, during, and after cultural shifts, such as the agricultural transition (Neolithic Revolution), the Industrial Revolution, and the advent of modern globalized and highly processed food (Adler et al., 2013). Studying these evolutionary snapshots provide information on how these communities have changed and adapted in real time, and improve our understanding of how these changes have influenced human health and disease.

In recent years, ancient DNA (aDNA) research has unveiled genomes of archaic hominins, such as Neanderthals (Prüfer et al., 2014), Denisovans (Meyer et al., 2012) as well as prehistoric human populations (Mathieson et al., 2015). Ancient DNA refers to damaged DNA extracted from fossil remains, and is characterized by short fragment sizes, cross linkage between DNA molecules, and increased cytosine deamination due to the taphonomic effects of the environment and time (Hofreiter et al., 2001; Willerslev and Cooper, 2005; Linderholm, 2015). Typical samples for extraction of aDNA are obtained from locations in the human body that offer the most protection from exogenous DNA (i.e., that from both the environment and microbes). The samples that have been most successful for the retrieval of hominin aDNA are bones (femur and petrosal) (Meyer et al., 2014; Pinhasi et al., 2015) and teeth (Adler et al., 2011).

While the vast majority of research in this field has focused on the extraction and analysis of genomic data for human population history, the last 5 years has seen cutting-edge research expose the potential of ancient human microbiome DNA (Adler et al., 2013; Warinner et al., 2015). This has primarily been done using dental calculus, a calcified bacterial biofilm (dental plaque) that forms on teeth. Due to the process of dental calculus formation that encapsulates microbial DNA protecting it from external contamination, it is possible to extract large amounts of microbial aDNA that are relatively uncontaminated by the environment. Dental calculus research has already provided insights into changes in the oral microbiome during large cultural shifts in time, including transitions to agriculture and industrialization (Adler et al., 2013).

Research to analyze the microbial diversity within dental calculus began with de la Fuente et al. (2013) using species-specific PCR amplification, followed by Adler et al. (2013) using 16S metabarcode sequencing and Warinner et al. (2014) using metagenomic shotgun sequencing. Ancient dental calculus revealed how the human microbiome has adapted through time, as observed through 34 ancient calculus samples in Europe over the past 8,000 years (Adler et al., 2013). The authors were able to show that both the transition to agriculture (∼7,500 years ago) followed by the availability of processed food, antibiotics, and toxins in the environment following the Industrial Revolution (∼150 years ago) resulted in decreased bacterial diversity and an increase in oral microbiota dysbiosis.

aDNA has also been used to explore the evolutionary history of specific microorganisms, such as the causative agents of tuberculosis (Bos et al., 2014), plague (Bos et al., 2011), leprosy (Inskip et al., 2015), and gastritis (Maixner et al., 2016). While important, these studies have not revealed how the microbiome in these individuals has shifted. Microbiome-based studies, using aDNA from dental calculus of ancient populations, can provide us with a unique perspective on the evolution of entire bacterial communities, and their coevolution with their hosts, rather than individual taxa (Adler et al., 2013; Metcalf et al., 2014; Warinner et al., 2014). The growing availability of genomic information from ancient humans (Haak et al., 2015; Mathieson et al., 2015; Skoglund et al., 2015) provides a unique opportunity to examine co-evolutionary interactions through time. For example, several studies have described the human genomic changes that occurred during the agricultural transition, including alterations in both immune and metabolic genes that allowed early farming communities to adapt to new diets and environments (Allentoft et al., 2015). These loci, such as those relating to lactase persistence and immune responses, are similar to loci identified in modern day microbiome – GWAS studies Goodrich et al. (2016b), suggesting that there may be a link between genomic and microbiome alterations.

## Future Directions

In order to obtain a detailed understanding of these interactions, larger sample sizes and independent replication cohorts are required to confirm the associations that are detected. This will be beyond the singular capacity of many research laboratories and will require large collaborations, as seen in the medical genetics community where GWAS studies have been applied to specific human diseases. As the list of microbiome associated genetic loci grows, there is also the need for functional studies to understand the mechanisms that underlie genome–microbiome interactions. Gnotobiotic mice and human cell lines hold promise toward that aim and are part of the NIH Integrated HMP (iHMP/HMP 2.0), announced in 2014 as the successor to the HMP. The Integrated HMP has initiated the transition from high throughput screening to longitudinal and detailed mechanistic studies [Integrative Hmp (iHMP) Research Network Consortium, 2014]. In addition, studying the co-evolution of the genome and microbiome in ancient humans using ancient DNA will provide a comprehensive overview of the changes to the interaction over time. Further research will also require multidisciplinary expertise that is missing in recent publications. Microbiologists and population geneticists will need to work together with bioinformaticians to accurately design experiments, perform rigorous statistical tests, and analyze results. With these steps, a clear understanding of the interactions between our genome and the microbiome will open new avenues for numerous medical therapies. This research will truly bring us into the age of personalized medicine, with the ability to modify our microbiome in light of our genome, to prevent disease and maintain human health.

## Author Contributions

MA-A wrote the manuscript; LW and AC commented and edited the manuscript. All authors read and approved the final version for submission.

## Conflict of Interest Statement

The authors declare that the research was conducted in the absence of any commercial or financial relationships that could be construed as a potential conflict of interest.
